# Borehole Equilibration: Testing a New Method to Monitor the Isotopic Composition of Tree Xylem Water *in situ*

**DOI:** 10.3389/fpls.2020.00358

**Published:** 2020-04-15

**Authors:** John D. Marshall, Matthias Cuntz, Matthias Beyer, Maren Dubbert, Kathrin Kuehnhammer

**Affiliations:** ^1^Department of Forest Ecology and Management, Swedish University of Agricultural Sciences, Umeå, Sweden; ^2^Université de Lorraine, AgroParisTech, INRAE, UMR Silva, Nancy, France; ^3^IGOE, Umweltgeochemie, Technische Universität Braunschweig, Braunschweig, Germany; ^4^Department B2.3: Groundwater Resources and Dynamics, German Federal Institute for Geosciences and Natural Resources (BGR), Hanover, Germany; ^5^Ecosystem Physiology, University Freiburg, Freiburg, Germany; ^6^IGB Berlin, Landscape Ecohydrology, Berlin, Germany

**Keywords:** transpiration, continuous sampling, root water uptake, Craig-Gordon model, δ^18^O, δ^2^H, isotopic labeling, xylem water

## Abstract

Forest water use has been difficult to quantify. One promising approach is to measure the isotopic composition of plant water, e.g., the transpired water vapor or xylem water. Because different water sources, e.g., groundwater versus shallow soil water, often show different isotopic signatures, isotopes can be used to investigate the depths from which plants take up their water and how this changes over time. Traditionally such measurements have relied on the extraction of wood samples, which provide limited time resolution at great expense, and risk possible artifacts. Utilizing a borehole drilled through a tree's stem, we propose a new method based on the notion that water vapor in a slow-moving airstream approaches isotopic equilibration with the much greater mass of liquid water in the xylem. We present two empirical data sets showing that the method can work in practice. We then present a theoretical model estimating equilibration times and exploring the limits at which the approach will fail. The method provides a simple, cheap, and accurate means of continuously estimating the isotopic composition of the source water for transpiration.

## Introduction

A large proportion of the precipitation falling into a forest ecosystem is returned to the atmosphere via evapotranspiration of trees and soils. The remainder eventually flows into streams and recharges groundwater. The returns can be divided into transpiration and evaporation, where transpiration refers to the uptake and loss of water from the interiors of plants and evaporation refers to all the rest, including evaporation from soils and plant surfaces (Jarvis and McNaughton, 1986). Finally, the responses to environmental conditions differ qualitatively and quantitatively between transpiration and evaporation; a satisfactory mechanistic description would therefore include separate controls over each component. Although hydrologists have traditionally lumped transpiration with evaporation, they show increasing interest in measuring and modeling the isolated fluxes (Jasechko et al., 2013; Evaristo et al., 2015; Dubbert and Werner, 2019). In contrast, ecophysiologists have long been interested in describing transpiration, in part because it links the carbon and water budgets of plants (Granier, 1987; Barr et al., 2007; Dubbert et al., 2013; Poyatos et al., 2016).

The transpiration flux can be distinguished from other evaporative fluxes by its unique stable-isotopic signature (e.g., Yakir and da Sternberg, 2000). In general, evaporating water undergoes two fractionations (Dongmann et al., 1974). The first, termed the equilibrium fractionation, is associated with the phase change, where the lighter isotope moves into the vapor phase somewhat more rapidly than the heavier one. The second fractionation occurs as the water vapor diffuses away from the evaporating surface. Termed the kinetic fractionation, it likewise favors the lighter isotope. Integrated over longer time intervals (>days), the isotopic signature of transpired water vapor occurs at steady-state, which means that the isotopic signature is equal to its water source and also equal to the signature of water transported in the xylem. This makes it easily distinguishable from strongly depleted soil evaporation (e.g., Dubbert et al., 2013). However, environmental conditions change so quickly that transpiration at isotopic steady-state is disrupted frequently over the diurnal course and environmentally stable periods are too short for leaves to return to isotopic steady-state (e.g., Simonin et al., 2013; Dubbert et al., 2014). Consequently, the isotopic signature of transpired water vapor follows a distinct diurnal pattern, being most depleted compared to source water during the morning hours, approaching isotopic steady-state throughout the day and being strongly enriched compared to its water source during night (e.g., Dubbert et al., 2014).

The source for transpired water is the water taken up by the roots and transported through the tree xylem. In contrast to the strong fractionation that occurs during transpiration, xylem water is generally taken up and transported without fractionation (Wershaw et al., 1966), though a growing number of exceptions are reported (Lin and Sternberg, 1993; Ellsworth and Williams, 2007; Vargas et al., 2017; Barbeta et al., 2019; Poca et al., 2019). One might expect that the xylem water would be identical to precipitation, but precipitation varies over the year, creating depth profiles in the soil. Moreover, many trees have access to groundwater, which may originate elsewhere (Busch et al., 1992; Beyer et al., 2018). Hence, xylem water isotopic composition is of particular interest for root water uptake studies (Schulze et al., 1996; Moreira et al., 2000; Kulmatiski et al., 2010; Rothfuss and Javaux, 2016) and when investigating hydraulic redistribution (Priyadarshini et al., 2016; Rothfuss and Javaux, 2016; Sprenger et al., 2016). Partitioning of evapotranspiration into its evaporation and transpiration components requires knowledge of the isotopic composition of the two components. Transpiration has been measured by collecting water that evaporates directly from the leaves, both as condensate in bags (Beyer et al., 2016) and as water vapor in chamber outflows (Haverd et al., 2011). In addition, one can also derive the isotopic signature of transpired vapor, either by assuming isotopic steady state (xylem water = transpired water vapor isotopic signature) or modeling transpired water vapor isotopic signature allowing for isotopic non-steady state (e.g., Dongmann et al., 1974; Cuntz et al., 2007; Farquhar et al., 2007).

There is a long tradition of describing the isotopic composition of xylem water via cryogenic distillation of the water held in plant xylem (White et al., 1985; Flanagan et al., 1992). The prerequisite for this method is the completeness of the extraction, which minimizes opportunities for isotopic fractionation between the original sample water and the resulting collected water. However, the approach has several weaknesses. First, it simultaneously distills other volatile organic compounds, especially from leaves (Martín-Gómez et al., 2015). These organic compounds are of little concern if the samples are analyzed by IRMS, but they are problematic in analyses using laser-based absorption techniques (West et al., 2010, 2011). The isotopolog-specific water peaks may be interfered with, especially by alcohol peaks. A second problem is that cryogenic extraction may extract water that is not moving in the xylem, for example water held in cell walls (Barbeta et al., 2018) and heartwood (White et al., 1985; Busch et al., 1992). In these cases, it would not represent the correct uptake source. At best, cryogenic extraction is laborious, slow, and can only represent a point in time (Koeniger et al., 2011). Other methods have been used for extracting soil water (Sprenger et al., 2016), but many of these are ineffective at the low water potentials typical of plant xylem.

When laser-based systems appeared on the market, it became possible to monitor the isotopic composition of water vapor continuously. It was soon recognized that this not only allowed for direct measurements of the isotopic signal of ambient, transpired and evaporated water vapor but also the continuous measurement of liquid water in soils and stems. This can be done by calculating the liquid isotopic composition from vapor phase values assuming equilibrium fractionation (Majoube, 1971) and requires recording temperature and a means of preventing condensation between the equilibration site and the instrument. This led first to several devices for *in situ* studies of soil water isotopes (Herbstritt et al., 2012; Soderberg et al., 2012; Rothfuss et al., 2013; Volkmann and Weiler, 2014), and then to a modification for tree xylem (Volkmann et al., 2016). The xylem system was able to detect unequivocally the arrival of a tracer pulse in the xylem water. However, it had difficulty matching the predicted isotopic composition of δ^18^O at natural abundance. It was inspiring as proof-of-concept, but it was worrisome for its complexity and the unexplained offset in the δ^18^O data.

**Figure 1:**
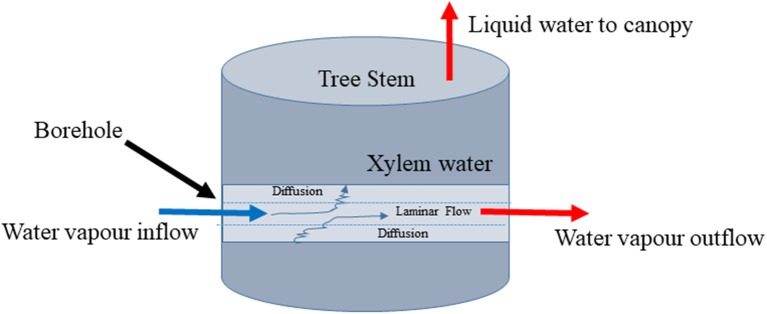
Conceptual diagram of stem borehole technique for measuring xylem water stable isotopic composition by equilibration.

We supposed that the problem might be simplified if only the liquid water in the xylem would continue to evaporate into a flowing airstream that passed through the stem of the tree. The isotopic effects might be particularly simple if we could ensure that the airstream reached isotopic equilibrium with the xylem water as it passed through the stem (Figure 1). To test this idea, we conducted two experiments and developed a model describing the isotopic equilibration of borehole vapor with liquid xylem water. The experiments tested whether the method was capable of returning the expected values. The model tested different assumptions about the physical processes behind the method and was used to explore its practical limits.

## Methods and Materials

### Cut-Stem Greenhouse Experiment

A preliminary test of the technique was conducted in a greenhouse at the Swedish University of Agricultural Sciences (SLU) in Umeå, Sweden in March, 2014. This experiment was artificial in the sense that a cut stem is unnatural, however, the power of an experiment is always that it controls some sources of variation. In this case, it allowed us to provide a known water source directly into the xylem at a precisely known point in time and at a precisely known distance from the borehole. A field-grown Scots pine (*Pinus sylvestris* L.) tree was cut near Vindeln, in northern Sweden. In the glasshouse it was recut underwater (to avoid cavitation) and, with the crown intact, the cut end was placed several centimeters below the water surface in a bucket containing liquid water of known isotopic composition. The water sources were exchanged for the next several days while the stems transpired and sucked the water up into the xylem. A borehole 6 mm in diameter was drilled through the stem ~30 cm vertically from the cut end, where the tree diameter was 12 cm. The borehole was rinsed with acetone to reduce pitch production from the resin canals inside. Because the acetone is both volatile and soluble in water, it was rapidly removed from the system. Apart from removing and blocking pitch production, it had no other obvious effects on the tree. The borehole was then plumbed into a Picarro L2130-i cavity ring-down absorption spectrometer (CRDAS) by screwing the pipe threads of a Swagelok connector into the borehole and attaching FEP Teflon tubing of 6 mm outside diameter (Swagelok, Solon, OH, USA) to the other side. The CRDAS analyzed the concentration and isotopic composition of water vapor in the air that had passed through the borehole. It was calibrated against known liquid water standards injected to the evaporator system at the beginning and end of the experiment. A copper-constantan thermocouple was inserted through the tubing and the fitting and placed in contact with the xylem inside the borehole, monitoring xylem temperature continuously throughout the experiment, recorded by a datalogger (CR10X, Campbell Scientific, Logan, USA).

As noted above, the water in the bucket was replaced approximately daily with water of a different isotopic composition to create cycles in the isotopic composition of the xylem water. The deionized Umeå tap water source had δ^2^H = −177 to −181 %0 and δ^18^O of −22.1 to −22.9 %0. This can be considered the control. The enriched source had δ^2^H −93.5 %0 and δ^18^O +8.1 %0; it was produced by evaporating deionized Umeå tap water to approximately 3% of its original volume. The bucket water was analyzed on an IRMS (Thermo-Finnigan details, SLU stable isotope lab, Umeå, Sweden) to check its composition at the end of each cycle. Sample lines were removed from the tree whenever condensation was visible, which occurred only in the morning when greenhouse temperatures were coolest. In this case, the lines were dried with air drawn through a tube filled with drying agent until the CRDAS water-vapor concentration and isotopic composition indicated that condensed water had completely evaporated.

The mean arrival time of the label was estimated from the midpoint of the breakthrough curves. The midpoint was calculated as the mean of the steady-state values at the beginning and end of the cycle. This analysis was done on the first cycle because it was the only one that occurred entirely in daylight, meaning that transpiration continuously moved the water up the stem during the breakthrough period. The other cycles included periods of twilight or darkness and so presumably delayed the arrival of the label. The arrival time was compared to estimates of sap flow velocity for Scots pine trees in the field and from the same area. It is unfortunate that we neglected to measure the water uptake rates directly from the buckets; this limitation partially motivated the second experiment.

### Intact-Root Greenhouse Experiment

The second greenhouse experiment was conducted in the Institute for Ecosystem Physiology, Freiburg, Germany in November and December 2018. One pine tree (*Pinus pinea* L.) was placed in a large pot under artificial light (12 h light, 12 h dark, switching on automatically at 07:00 o'clock). Two stem boreholes, positioned in parallel, were drilled through the trunk at 15 and 65 cm stem height, respectively. The boreholes were 10 mm in diameter and passed through the entire stem. Trunk diameter was 9.9 cm at the lower borehole and 8 cm at the upper one. Boreholes were flushed, as in the first experiment, with Acetone (Rotipuran, ≥ 99.8%, Carl Roth, Germany). Commercially available Swagelok connectors were then inserted at both sides of each borehole and PTFE tubing was attached.

**Figure 2:**
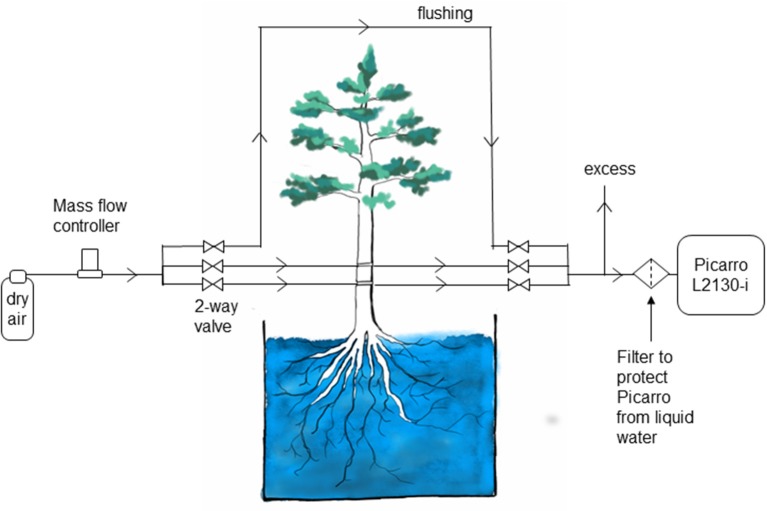
Schematic of the setup for measuring water stable isotopes in tree xylem *in situ* in the Freiburg experiment.

Measurements in different boreholes as well as a control water vapor standard and a flushing line were combined in one automatic system. Automatic switching between individual measurements was realized with solenoid magnetic valves (2-Way Elec. Valve, EC-2-12, Clippard Minimatic, USA). For each measurement, a pair of valves (one at the inflow, one at the outflow) was opened and 80 mL min^−1^ of dry air, regulated via a mass flow controller (FC 260, Tylan General TCA GmbH, Germany), was pushed through the selected borehole or flow path. Water vapor was exchanged at areas with contact to liquid water and sampled gas was directed into a cavity ring-down spectroscopy analyzer (L2130-I, Picarro Inc., USA) to determine its water vapor isotopic composition. Figure 2 shows a schematic drawing of the experimental setup.

In this experiment, we submerged intact tree roots in water of known isotopic composition (δ^18^O = −8.43 ± 0.05 %0, δ^2^H = −59.28 ± 0.24 %0). We included the roots in this second experiment out of concern for the impact of soil evaporation and potential isotopic fractionation due to the soil matrix (Orlowski et al., 2013, 2016; Gaj et al., 2017), which might therefore cause isotopic heterogeneity across the root system. This also had the advantage of enabling regular, uncomplicated sampling of the tree's source water. To avoid anoxic conditions, the water was well aerated using mini-pumps. On 21 November we changed the tree's source water to ^2^H_2_O labeled water (δ^18^O = −9.22 ± 0.19 %0, δ^2^H = +297.57 ± 3.08 %0) and monitored the subsequent change of the isotopic composition in the tree xylem at high time resolution. Weekly refilling to replace transpired water impacted source water isotopic composition. Samples were collected before and after each refilling. The isotopic composition of source water samples was measured with the same laser used for *in situ* measurements but using a vaporizer and switching the measurement mode to liquid injections.

Temperature and relative humidity (RH) of ambient air were recorded every 30 min (OM-EL-USB-2-PLUS, Omega Engineering Inc., United States, accuracy: RH: 2%, T: 0.3°C). Sap flow velocity was estimated with a sap flow sensor (heat pulse velocity sensor, Edaphic Scientific, Australia), installed 20 cm above and perpendicular to the upper borehole. After installation the sensor was thermally insulated. A baseline value for a sap flow velocity of 0 was determined after the experiment by inserting the sensor into a tree without leaves. For calculating sap flow velocities the method described in Hogg et al. (1997) was used.

### Data Processing

Individual measurements lasted for 15 min and were preceded by a 5 min period of flushing the valve system with dry air. This on the one hand removed water vapor of the previous measurement from the tubing, while on the other hand allowed for detection and (to some extent) removal of condensed water. Mean values for each measurement were calculated over the last 3 min of each cycle, when values of measured water vapor and isotopic composition were stable. Data points with a standard deviation greater than 0.5 %0 and 1.5 %0 for δ^18^O and δ^2^H raw values, respectively, were excluded from the data set. This was true for 2.4% of all measurements. On average boreholes were sampled every 3 h.

Temperature (*T*, °C) was recorded every minute with Type-T thermocouples (copper-constantan) in the bottom borehole and used to calculate equilibrium fractionation (Majoube (1971), as well as saturation vapor pressure inside the borehole (Murray, 1966). The water vapor concentration measured by the cavity-ringdown spectrometer was then compared to the saturated specific humidity at stem temperature *T* to obtain an estimate of relative humidity in the borehole. The relative humidity was used to assess whether full saturation with water vapor was achieved inside the borehole, indicating whether the assumption of equilibrium conditions inside the borehole was valid. It also revealed potential condensation under the given environmental conditions. Ideally, relative humidity *h* should approach 1.0. We arbitrarily chose an *h* of 0.8 as an indication that the dry air pushed into the borehole did not approach saturation. Data points with relative humidity below 0.8 were therefore excluded from the final, processed data set. Thus, excluded were 0.7 and 2.4% of the measurements from the bottom and top borehole, respectively.

Standardization of isotope measurements was done by manually sampling the headspace in four water vapor standards, once per day in the morning. The standards were held in airtight coffee bags (WEBAbag CB400-420BRZ, Weber Packaging GmbH, Germany) each filled with 50 ml of isotopically distinct water with −14.9, −9.4, 3.4 and −9.2 %0 δ^18^O and −110.8, −66.3, 3.8 and 367.7 %0 δ^2^H, for light, medium, heavy, and label standard, respectively, and inflated with dry air. After each sampling, bags were refilled with dry air and kept in an insulated box, in which temperature was recorded throughout the day. Analogously to borehole measurements, liquid isotopic compositions were calculated from measured vapor values and *T*, assuming thermodynamic equilibrium. For quality control, a water vapor standard in an airtight glass container (Duran 1,000 ml, Schott AG, Germany) was integrated into the system and its headspace was automatically sampled (every 2–3 h). After processing collected data in the same way as the other measurements, derived isotopic compositions were 0.61 and 1.12 %0 lighter compared to reference values of liquid water inside the bottle for δ^18^O and δ^2^H, respectively.

### Model Description

The model describes the isotopic composition of water vapor in air as it leaves the borehole and flows toward the isotope analyzer. It describes air flowing through a borehole of radius *r* in a tree stem of diameter *l*. The air inside the borehole of Volume *V* = π*r*^2^*l* is turned over once after the time *t*_u_ = *V*/*u*_*o*_. If the flow rate *u*_o_ is given in mol/s, then *V* has to be divided by the molar volume *V*_1mol_ = 22.414/1000·*T*/*T*_0_·*p*_0_/*p* to give $\tilde{V}$ = *V*/*V*_1mol_, with standard temperature *T*_0_ = 273.15 K and standard pressure *p*_0_ = 101 325 Pa.

We want to estimate whether this turnover time *t*_u_ is sufficient to allow full equilibration of incoming air with the water around the borehole walls. If the airflow is laminar, air is transported by molecular diffusion from the airstream to and from the borehole walls. The maximum diffusion time *t*_*d*_ is hence:

(1) $$\begin{array}{l} t_{d}=\frac{r^{2}}{4D} \end{array}$$

with the diffusivity of vapor in air *D* = *D*_0_·*p*_0_/*p*·(*T*/*T*_0_)^1.88^ and *D*_0_ = 2.12·10^−5^ m^2^/s. The velocity profile within the borehole is then parabolic with maximum velocity in the center of the air stream being twice the mean velocity, which is the measured velocity *u*_*obs*_. We take hence *u*_*o*_ = 2*u*_*obs*_ during the rest of the manuscript. The turnover time of the borehole volume by the air stream is then:

(2) $$\begin{array}{l} t_{u}=\frac{\tilde{V}}{2u_{obs}} \end{array}$$

By comparing *t*_*u*_ and *t*_*d*_, we can get an indication of whether the passage of air through the borehole is sufficiently slow to allow diffusion, which requires time *t*_*d*_, to occur into the central air stream before the air stream leaves the borehole, which requires time *t*_*u*_. Ideally, *t*_*u*_ would be much greater than *t*_*d*_.

#### Total Water Vapor in the Borehole

We next sought to determine whether the borehole water vapor concentration would reach saturation before leaving the borehole. We note that air enters the borehole with flowrate *u*_*i*_ and mole fraction *w*_*i*_ and leaves the borehole with flowrate *u*_*o*_ and mole fraction *w*_*o*_. If *u*_*o*_ is measured on moist air, then the flow rate is the same as the incoming flow rate *u*_*i*_. If *u*_*o*_ is measured on dry air, then the incoming air flow must be corrected by the different vapor mole fractions of incoming and outgoing air: *u*_*i*_ = *u*_*o*_(1–*w*_*o*_)/(1–*w*_*i*_) (von Caemmerer and Farquhar, 1981). The change of the mole fraction within the borehole is hence the difference between incoming and outgoing moist air flow plus any evaporation *E* from the borehole surface *A*:

(3) $$\begin{array}{l} \tilde{V}\frac{dw_{o}}{dt}=u_{i}w_{i}-u_{o}w_{o}+\tilde{A}E \end{array}$$

with $\tilde{A}$ = *A*/*V*_1mol_. If we assume that the surface of the borehole is not drying out, meaning that water is continuously supplied to the surface, then *E* is the exchange flux between the water vapor partial pressure above the borehole surface at stem temperature *T*_*s*_ and the air stream. The exchange flux happens by molecular diffusion *E* = *D*·*dw*/*dr*, which gives:

(4) $$\begin{array}{l} \tilde{V}\frac{dw_{o}}{dt}=u_{i}w_{i}-u_{o}w_{o}+\frac{\tilde{A}D}{r}\left(w_{sat}-w_{o}\right) \end{array}$$

with *w*_*sat*_ = *e*_*sat*_(*T*_*s*_)/*p* and the saturation partial pressure over liquid water *e*_*sat*_. Note that *A*/*r* = 2π*l*., i.e. independent of the radius of the borehole *r*.

If *u*_*i*_ = *u*_*o*_, this gives the non-steady-state development of the water vapor mole fraction within the borehole as:

(5) $$\begin{array}{l} w_{o}\left(t+dt\right)=w_{o}^{ss}+\left(w_{o}\left(t\right)-w_{o}^{ss}\right)e^{-\frac{dt}{t_{w}}} \end{array}$$

with the steady-state mole fraction:

(6) $$\begin{array}{l} w_{o}^{ss}=\frac{u_{o}w_{i}+\frac{\tilde{A}D}{r}w_{sat}}{u_{o}+\frac{\tilde{A}D}{r}} \end{array}$$

and the time constant:

(7) $$\begin{array}{l} t_{w}=\frac{\tilde{V}}{u_{o}+\frac{\tilde{A}D}{r}} \end{array}$$

The steady-state mole fraction in the borehole is hence a weighted mean of the incoming mole fraction and the vapor coming from the borehole surface. Both airflow and diffusion help to reach steady state (Equation 6).

#### Isotopic Composition of Total Water Vapor in the Borehole

All mole fractions in Equation (7) carry an isotopic signature. There is a kinetic fractionation α_*k*_ (>1) during diffusion, but laminar airflow does not fractionate. This leads to the corresponding differential equation for the development of the isotopic composition in the borehole:

(8) $$\begin{array}{l} \tilde{V}\frac{dw_{o}R_{o}}{dt}=u_{i}w_{i}R_{i}-u_{o}w_{o}R_{o}+\frac{\tilde{A}}{r}\frac{D}{\alpha_{k}}\left(w_{sat}R_{s}-w_{o}R_{o}\right) \end{array}$$

If the isotopic composition of the vapor partial pressure above the borehole surface *R*_*s*_ is in isotopic equilibrium with xylem water *R*_*s*_ = *R*_*x*_/α^+^, and *u*_*i*_ = *u*_*o*_, then the non-steady state development of the isotopic composition of water vapor in the borehole writes equivalent to Equations (5–7):

(9) $$\begin{array}{l} R_{o}\left(t+dt\right)=R_{o}^{ss}+\left(R_{o}\left(t\right)-R_{o}^{ss}\right)e^{-\frac{dt}{t_{x}}} \end{array}$$

with the steady-state mole fraction:

(10) $$\begin{array}{l} R_{o}^{ss,x}=\frac{u_{o}w_{i}R_{i}+\frac{\tilde{A}D}{r}w_{sat}\frac{R_{x}}{\alpha_{k}\alpha^{+}}}{u_{o}w_{i}+\frac{\tilde{A}D}{r}\left[w_{sat}-\left(1-\frac{1}{\alpha_{k}}\right)w_{o}\right]} \end{array}$$

and the time constant:

(11) $$\begin{array}{l} t_{x,x}=\frac{\tilde{V}w_{o}}{u_{o}w_{i}+\frac{\tilde{A}D}{r}\left[w_{sat}-\left(1-\frac{1}{\alpha_{k}}\right)w_{o}\right]} \end{array}$$

where the index *x* indicates the assumption *R*_*s*_ = *R*_*x*_/α^+^. It can be shown that *t*_*x, x*_ ≥ *t*_*w*_ and *t*_*x, x*_ = *t*_*w*_ if α_*k*_ = 1. Note that only α_*k*_ = 1 gives a steady-state isotopic composition, which is a simple weighted mean of the isotopic compositions of incoming air and xylem water.

*R*_*s*_ = *R*_*x*_/α^+^ assumes that water at the borehole surface is not changing but staying constant at the isotopic composition of xylem water *R*_*x*_, even when exchanging isotopically with vapor in the borehole. If the borehole is not drying out, there must be a supply of water to the borehole surface. The water at the borehole surface might hence tend to a steady-state isotopic composition, where the evaporated water has the isotopic composition of the supply water, similar to leaf water enrichment (Craig and Gordon, 1965). Assuming that supply water has the xylem isotopic composition *R*_*x*_, this means that the last term of Equation (11) is replaced by $\tilde{A}$*ER*_*x*_ and the steady state composition is:

(12) $$\begin{array}{l} R_{o}^{ss,C}=\frac{u_{o}w_{i}R_{i}+\tilde{A}ER_{x}}{u_{o}w_{i}+\tilde{A}E} \end{array}$$

and the time constant:

(13) $$\begin{array}{l} t_{x,C}=\frac{\tilde{V}w_{o}}{u_{o}w_{i}+\tilde{A}E} \end{array}$$

with *E* given in Equations (6) and (7), and the index *C* indicating the Craig and Gordon steady-state assumption.

#### Water Vapor and Its Isotopic Composition Along the Borehole

The above derivations assume well-mixed borehole air, which is opposite to the idea of laminar flow. There will rather be a progressive development of water vapor saturation and then isotopic equilibrium from the inflow of the borehole to the outflow of the borehole. Each borehole segment passes on water vapor to the next after interacting with the water on the borehole walls. This would lead to a 2D advection-diffusion equation similar to Equation (20) of Ogée et al. (2007). We do not attempt to solve such a partial differential equation here. We rather discretize the borehole in *N* segments along the air flow path, where the outflow of one segment is the inflow of the next segment, i.e. that *w*_*o*_ and *R*_*o*_ of segment *i*−1 is *w*_*i*_ and *R*_*i*_ of segment *i*. Such a procedure approaches the exact solution when using small segments, i.e., large *N*.

We calculate in the following only solutions to the steady-state equations, assuming rather steady conditions of incoming air, i.e., *w*_*i*_ and *R*_*i*_. We note that atmospheric concentrations near a forest floor can change rapidly due to emanation from or exchange with the ground. Such rapid fluctuations can be alleviated using a buffer volume. We hence present the following calculations assuming steady state conditions in vapor isotopic composition in all *N* segments.

We further differentiate three cases:

(3.1) constant isotopic composition of borehole surface water, *R*_*s*_ = *R*_*x*_/α^+^,
(3.2) Craig-Gordon steady state for the water at the borehole surface, *R*_*s*_ = *R*_*C*_/α^+^, where the borehole surface is in steady state with the vapor isotopic composition of the current segment, and
(3.3) a mixture of both, assuming mixing of unenriched xylem water with the evaporating water at the borehole surface, *R*_*s*_ = *f R*_*x*_/α^+^ + (1–*f* )*R*_*C*_/α^+^.

The model cases are used to assess the isotopic composition across a range of conditions. These include variations in flow rate, tree diameter, incoming water vapor concentrations, and fractional mixing of xylem water with water in isotopic equilibrium with borehole vapor.

## Results

### Cut-Stem Greenhouse Experiment

**Figure 3:**
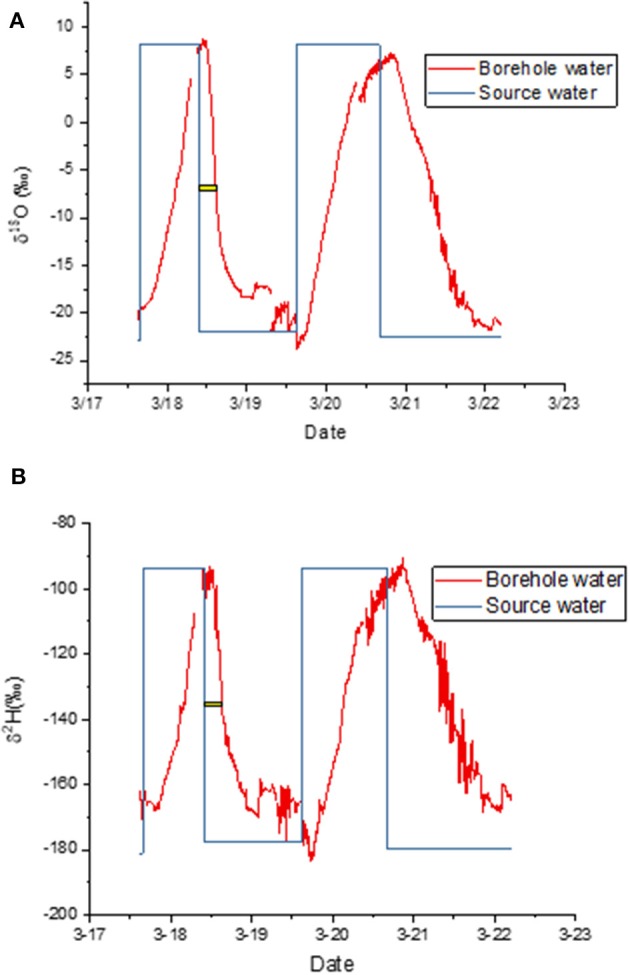
**(A,B**) Measured isotopic composition of water vapor exiting the borehole (red) and water vapor composition predicted from source water, i.e., source water isotopic composition plus equilibrium fractionation (blue). The yellow bar shows the lag between the change in source water and the mean arrival time in the borehole. Panels show time courses for **(A)** δ^18^O and **(B)** δ^2^H.

When the label was taken up by a cut stem, the asymptotic values at the end of each labeling cycle confirmed that the measured isotopic composition matched the expected values well (Figures 3A,B). It took several hours after a change in water source before this agreement was reached because it took several hours for the label to reach the borehole. The yellow bars in Figure 3 show the time elapsed between the change in source water and the time when the change in label intensity reached its midpoint. We use that time as an estimate of the average arrival time for the label. Only the first cycle was used because it was entirely during the light period, meaning that transpiration continued throughout this period. We estimate that the decline required 4:55 h for δ^18^O and 5:21 for δ^2^H to reach this inflection point.

There was evidence of condensation in the early morning during this first experiment (Figure 3). We therefore disconnected the lines each morning and attached a Drierite column to the borehole inlet. We then pumped dehumidified air through the tubing until the water vapor concentration fell to nearly zero. This dry-down was accompanied by a typical Rayleigh distillation curve as the residual water evaporated (not shown).

### Whole-Root Greenhouse Experiment

**Figure 4:**
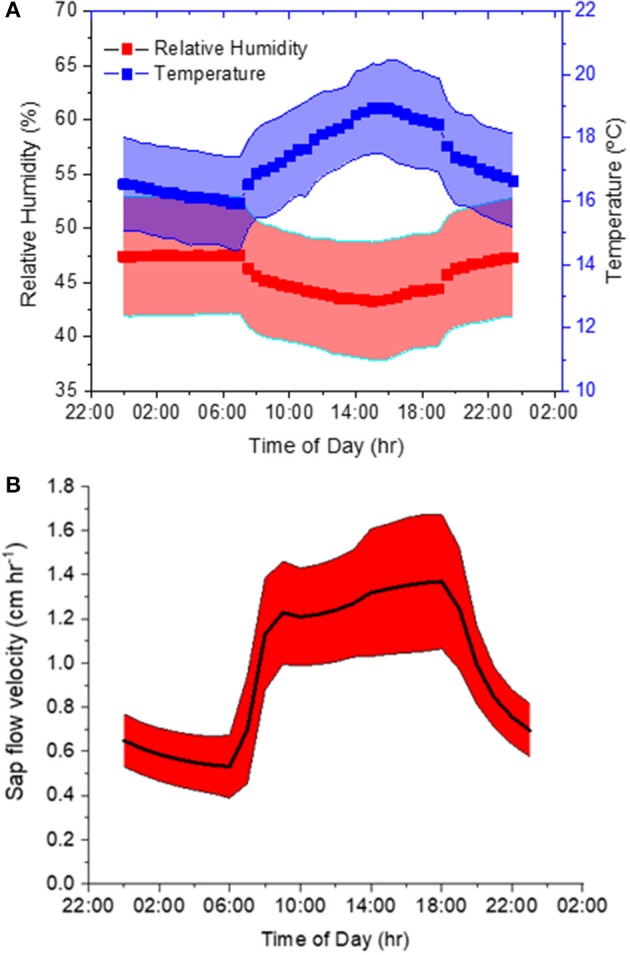
**(A,B)** Diurnal variations of **(A)** ambient temperature (T) and relative humidity (RH), as well as **(B)** sap flow velocity averaged over the six-week course of the experiment.

Our second experiment tested the method using trees with intact root systems. During this experiment, ambient air *T* and *RH* averaged 17.3 ± 1.7°C and 46 ± 6% and featured a pronounced diurnal course corresponding to the prevalent light cycle (Figure 4A). The variation was large because these diurnal curves represent diurnal patterns over 6 weeks in an indoor hangar not designed to maintain constant conditions. T measured inside the borehole was on average 16.8 ± 1.3°C and therefore 0.5°C lower than the ambient air. Sap flow had a mean velocity of 0.97 ± 0.39 cm h^−1^ and it showed a distinct daytime increase. Mean values with standard deviations over the diurnal course are displayed in Figure 4B.

We used the model to estimate time constants for the borehole geometries, which are presented in Table 1. The much higher values of *t*_*u*_ compared to those of *t*_*d*_ or *t*_*s*_ again suggest that the air was inside the borehole long enough for the diffusion and evaporation to cause the water vapor in the borehole to approach equilibrium.

**Table 1:** Tree and borehole characteristics, as well as time constants for air flow and diffusion.

**Borehole**	**Height**	**Tree diameter**	***t_***d***_***	***t_***u***_***	***t_***w***_***
Top	65 cm	8 cm	0.24 s	1.77 s	0.46 s
Bottom	15 cm	9.9 cm	0.24 s	2.33 s	0.47 s

*t_d_ is the time constant for diffusion across the unstirred layer at the edge of the borehole, t_u_ is the time constant for passage through the stem, and t_w_ is the time constant to reach water vapor saturation. These values suggest that the air passing through the borehole had adequate time to approach isotopic equilibrium*.

**Figure 5:**
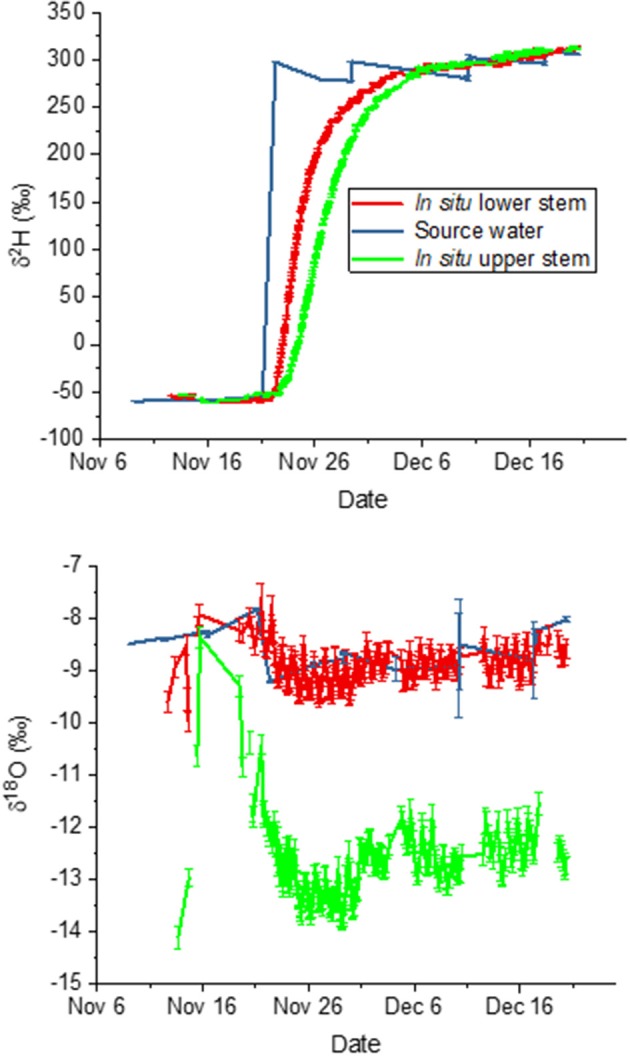
Time course of liquid xylem water isotopic compositions derived *in situ* from measured vapor phase values for the top and bottom borehole in relation to the tree's source water.

For this second experiment, we again compared measured isotopic composition to predictions based on source water values. We present the time courses of liquid xylem water isotopic composition in relation to the trees' source water in Figure 5. From these data, we calculated mean deviations of the *in situ* liquid xylem water measured from that of source water during periods that showed stable isotope values for δ^2^H. The measurements before 22 November represent natural abundance, those after 3 December 2018 show the effect of the label. A negative deviation signifies that *in situ* isotopic composition was more negative compared to source water. For the bottom borehole, the deviations were nearly zero. This means that the derived xylem water isotopic composition agreed with the source water values according to the model assumption 3.1 for both natural abundance (δ^18^O = −0.1 ± 0.6 (SD) %0, δ^2^H = 1.8 ± 2.3 %0) and the label phase (δ^18^O = −0.25 ± 0.22 %0, δ^2^H = 0.09 ± 7.8 %0). In contrast, the top borehole showed systematic deviations from source water values for both δ^18^O and δ^2^H. δ^18^O xylem values were depleted in ^18^O in relation to source water by −2.8 ± 1.5 %0 and −3.9 ± 0.3 %0 for the natural abundance and label phase, respectively. The δ^2^H xylem values were more similar in ^2^H to source water, differing by only 5.3 ± 3.0 and 1.9 ± 8.5 %0.

In both boreholes δ^2^H increased in a similar pattern from initial source water values (natural abundance) to the labeled water value after the change in source water on 21 November. However, in the top borehole the increase was delayed and less steep. We compared the lags in arrival of the label to measured sap flow velocities. The velocity estimated by the sap flow probes was 0.97 ± 0.4 cm hr^−1^. If we estimate time to the midpoint in the δ^2^H increase over time, which provides an estimate of the average uptake velocity, we find it after 70 h at the lower borehole and 124 h at the upper one. The lag time between the two was thus 54 h, yielding 1.08 cm hr^−1^ on average. When compared to the mean sap flow velocity, the agreement is very good.

Although the model predicted that the borehole water vapor should be at saturation, we wished to confirm that the borehole air had reached water vapor saturation by the time it left the borehole. This was important because it might influence whether the water vapor had approached isotopic equilibrium. We therefore calculated relative humidity corrected to borehole temperature for every measurement (see Material and Methods section). On average relative humidity was 98 ± 2% for the bottom borehole and 88 ± 3% for the top borehole.

### Modeling

We used the model to test the feasibility and limits of the borehole method. We first estimated the time constants for diffusion of water vapor to and from the borehole wall, and for the flowing airstream in the center of the borehole. If the diffusion were much faster than the passage through the borehole, it would be reasonable to assume that the isotopic composition of the water vapor in the borehole reflects that of the xylem water on the walls. We began by assuming the size of the borehole (10 mm), the flow rate (40 mL min^−1^, the flow rate induced by the vacuum pump) and temperature (10°C), we found that the throughflow time (*t*_*u*_) was 5.9 s. The time for diffusion (*t*_*d*_) was 0.28 s, more than 20 times faster. This result supported the notion that the borehole water vapor could come almost to equilibrium with the xylem water during its passage through the stem.

The model describes the relative humidity in the borehole both at steady-state and non-steady state conditions (Equations 6–10). Using the parameter values above, and adding an inflow relative humidity of 50%, we estimated that relative humidity would reach 99% after thrice the time constant (*t*_*w*_ = 0.24 s) inside the borehole. Not surprisingly, *t*_*w*_ is almost the same as the *t*_*d*_ estimate above.

The model solves for the isotopic composition of water vapor in the airstream leaving the borehole when the water vapor was passed from one segment in the stem to the next. We assumed the xylem water had an isotopic composition of −15 %0 and that the incoming water vapor was −20 %0. The rapid increase in humidity described above is accompanied by diffusion to and from the walls of any water vapor in the laminar airstream. These vapor fluxes are small in comparison to the liquid water pool in the xylem, but they determine the isotopic composition of the thin rind of water that exchanges.

Recall that the model compared three opposing ideas about how this rind should behave. First (model assumption 3.1), we assumed that it would simply mix into the much larger volume of xylem water that surrounds it. Next (model assumption 3.2), we assumed that the thin rind would enrich with time, reaching a steady-state isotopic composition given by the Craig-Gordon equation. Finally (model assumption 3.3), we assumed enrichment of the thin rind to the Craig-Gordon value but also a constant recharge of new xylem water into the rind. We found that the mixing rate needed to equal or exceed 35% of Craig-Gordon rind water with the remainder as unenriched xylem water. We used this value for our assessments of assumption 3.3 in all the work that follows.

**Figure 6:**
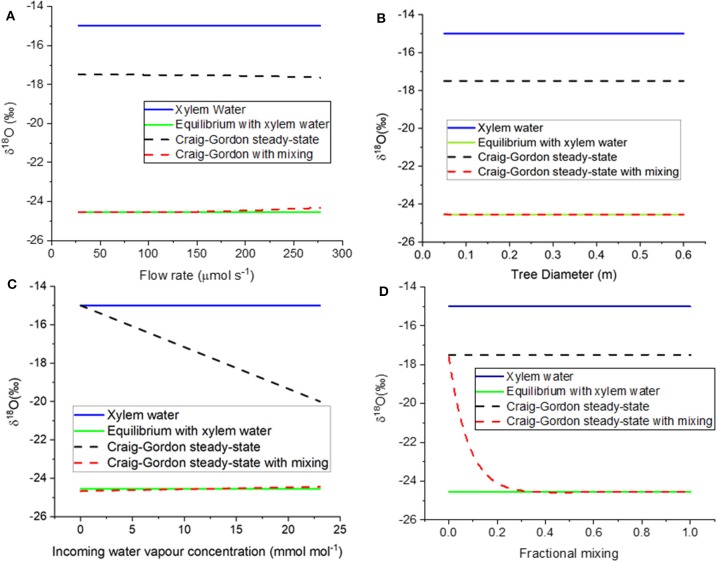
**(A–D)** Modeled δ^18^O of borehole xylem water. The figures show responses to variation in **(A)** flow rate through the stem, **(B)** stem diameter, and therefore wetted length of the borehole, **(C)** incoming water vapor concentration, and **(D)** fractional mixing of unenriched xylem water with borehole surface water in Craig-Gordon steady state. The four curves in each panel show the liquid stem water (blue solid line), water vapor in equilibrium with stem water (green solid line, model assumption 3.1), borehole surface water in Craig-Gordon steady-state (black dashed line, model assumption 3.2), and constant renewal of 35% of enriched borehole surface water with unenriched xylem water (red dashed line, model assumption 3.3).

The different assumptions are compared to model results in Figures 6A–D. When model assumption 3.1 (equilibrium with xylem water) was thus parameterized, the resulting value was −25.6 %0; this compares well with the equilibrium value of −25.7 %0. If the flow rate is defined by the CRDAS pump (40 mL/min, or 0.000028 mol s^−1^, at the far left of Figure 6A), then the steady-state with mixing water isotopic compositions was similar to the equilibrium value. Even if the flow were increased by ten-fold, the resulting steady-state prediction would still agree reasonably well. The Craig-Gordon steady-state value (model assumption 3.2) was quite different, but varied little, across the range of flow rates.

As tree diameter increased, the model predicted that water vapor concentration approaches saturation and the δ^18^O of water vapor approaches equilibrium with the xylem water. Already at diameter 0.05 m, the steady-state with mixing agrees with the equilibrium with xylem water (Figure 6B). Again, model assumption 3.2 (Craig-Gordon steady state) is nearly constant, but quite different from the other two assumptions.

We also used the model to test the influence of the relative humidity of the air entering the borehole (Figure 6C). The Craig-Gordon model (assumption 3.2) was the only one that predicted a difference in the isotopic composition of the vapor, decreasing the predicted δ^18^O linearly as the inlet humidity increased.

As noted above, model case 3.3 allowed for mixing of the unenriched xylem water with the evaporating water at borehole surface, where the latter was described by the Craig-Gordon equation. The x-axis in Figure 6D is a proportional mixing rate between the xylem water and the evaporating water. In the absence of mixing, the Craig-Gordon steady state (assumption 3.2) applies. But as the mixing rate increased, the water vapor from the mixing assumption (3.3) approached equilibrium with the unenriched xylem water (assumption 3.1). As noted above, in our modeling we used a value of 0.35. This was the lowest rate that yielded a value consistent with the empirical data, which approached equilibrium with the unenriched xylem water (Figures 3, 5). However, any value higher than 0.35 would yield the same result.

## Discussion

We present the results of two experiments and a modeling exercise that show the isotopic composition of xylem water can be continuously monitored with a borehole through the stem. Under these conditions, the borehole water vapor predicted the isotopic composition of the liquid water in the xylem. This was true of both natural abundance and labeled water sources. Moreover, the label arrived at the boreholes approximately when it was expected. Sap flux in Scots pine (the cut-stem experiment) is reported to occur at about 1 meter per 12-h day (Tor-ngern et al., 2017). Because the height of the borehole above the cut end of the stem was 0.40 m, theory suggests that the label should have arrived in 4.8 h. Thus, the prediction was also well matched by the observations. This means that the method was able to detect the shift in xylem water approximately when it was expected to arrive. Sap flow was measured in the intact root experiment and, as noted above, it matched the arrival times very well.

The model shows that the borehole water vapor pool approaches equilibrium with the xylem water under suitable conditions, including the ones we measured. We also show that the method should work up to rather high flow rates, in small stems, and in the presence or absence of incoming water vapor. If there were problems, they would likely be detected as a failure to reach water vapor saturation at stem temperature.

### Condensation and Water Scrubbing

The high relative humidity required by this method leads to practical concerns about condensation in the borehole and in the lines leading to the analyzer. This would create memory effects due to the condensed water, delaying the arrival of the isotopic signal from the xylem to the analyzer. This is one weakness of the borehole technique; condensation must be considered in the sampling design and frequent checks are needed to ensure that the sample lines are dry. However, this has been recognized as a main issue in any online water isotope measurement approach (e.g., Volkmann and Weiler, 2014; Gaj et al., 2016). We addressed this problem in the Umeå experiment by manually attaching a dry air supply to the lines every morning and evaporating the condensate. In the Freiburg experiment, an automated system provided dry air to the sample lines at intervals, clearing the lines of any condensate that might have formed. In addition, the sample lines were heated (Volkmann et al., 2016). The success of these approaches is reflected in the rapid response to changes in the xylem water supply, which matched predictions based on sap flow in both experiments. A second weakness of the approach is the risk of drawing liquid water into the CRDS. A mesh or filter can be used to protect against this. Ideally, the mesh would be small enough to block flow in the tube, stopping the water at the filter (Figure 2).

It may seem obvious that scrubbing the water out of the inlet air should improve the data. There would then be no need to make assumptions about the mixing of incoming water vapor and evaporated xylem water mixing. Any water in the exiting airstream would necessarily have come from the xylem, especially in the presence of positive pressure (Volkmann et al., 2016). The disadvantage is that the scrubbing of water vapor requires the replacement of chemical traps, or power to run a drying tower. Our model case 3.3 suggests that such precautions are unnecessary given the apparent mixing with xylem water (Figure 6C). The model results are confirmed by the match to empirical data even in the absence of water scrubbing (Figure 3).

### δ^18^O Discrepancies

Data from the Freiburg experiment showed an offset of up to 4 %0 in δ^18^O (for the time after the labeling pulse) relative to source water values in one of the boreholes. Interestingly, the borehole was more depleted than the source water, almost the same offset as found by Volkmann et al. (2016) in their equilibration probe experiments. No such discrepancy was observed in the Sweden data. The discrepancy observed in the Freiburg experiment can likely be attributed to non-equilibrium conditions caused by the shorter exchange path (smaller stem diameter) and was only observed in the upper of the two boreholes. Volkmann et al. (2016) speculated that their discrepancy was due to organic contamination of the water vapor, which interferes with the wavelengths used to determine the isotopic composition. They suggested that these interferents can be accounted for in post-processing. In the presented experiments, we did not find a major influence of contamination by organic compounds; otherwise source and measured isotopic compositions would not have matched (see West et al., 2010, 2011). We argue here that the Volkmann et al. (2016) offset might be due to non-equilibrium conditions as well.

One other possible explanation for the δ^18^O discrepancy in the intact-root experiment, but not the cut-stem experiment, is that the roots fractionate against ^18^O during water uptake. Such fractionation was detected in mangroves (Lin and Sternberg, 1993) and a range of woody xerophytes (Ellsworth and Williams, 2007), but it was detected for δ^2^H and not δ^18^O. This is opposite the pattern we observed, where the discrepancy was in δ^18^O, not δ^2^H. We therefore think that fractionation upon root uptake is not responsible for the observed discrepancy.

### Location of Equilibration

What is the actual surface area equilibrated? The model (Figure 6B) assumes that the entire surface area of the borehole is available for equilibration. However, a core of heartwood at the center of a tree stem is often dry. If it is dry, its thickness should be removed from the calculated area. This may be a significant loss in some stems, e.g., oak trees. If it is not dry, as when the heartwood is partially decayed, its isotopic composition may be different from the sapwood. In fact, such differences have seldom been observed when the heartwood has been checked. However, if the heartwood signal were different, it might carry through into the outlet airstream, especially if the sapwood is thin (Turner et al., 2000).

This leads to the question of which part of the stem actually controls the final isotopic composition. Given the rapid equilibration and slow airflow in our experiment, it would seem that the final composition would be determined in the last few cm before the airstream is carried out of the tree. We used the pipe-fitting threads on Swagelok fitting to attach tubing to the boreholes. These fittings were turned approximately two cm into the tree. The outer portion was bark, which should be excluded from the xylem equilibration. However, the inner portion entered the sapwood and blocked contact between the borehole airspace and the outermost sapwood. This would also reduce the borehole surface area and might, if the sapwood were narrow, prevent equilibration. Sapwood thickness varies greatly amongst species (Marshall and Waring, 1986; Turner et al., 2000; Quiñonez-Piñón and Valeo, 2017), but it was thick in our pines—nearly to the center (as also observed by Tor-ngern et al., 2017). The good match of source to measured water isotopic composition suggests that in the present case this was not an issue. Furthermore, the model predicts that equilibration occurs even over distances as short as 0.05 m (Figure 6B). On the other hand, the data from the upper borehole in the Freiburg experiments recommend caution when using stems less than 10 cm in diameter if the flow rate is 80 mL min^−1^ or more. In summary, the design of a stem borehole study needs to consider sapwood thickness depending on the species-of-interest and its growing conditions.

### Pitch and Cavitation

The borehole creates a significant wound in the stem, especially if it is as large as the ones we used (6 and 10 mm diameter in Umeå and Freiburg, respectively). The model suggests that the diameter has no effect on the equilibration, but pines tend to produce pitch around wounds in the stem, which might close the hole. Before data collection, we washed the boreholes repeatedly with acetone hoping that it would remove the pitch and kill the cells that would otherwise produce more. This appeared to keep the boreholes open, but there was still some pitch appearing on the borehole walls. We presume that these wall deposits might further decrease the effective area available for equilibration. This problem should be reduced in most other genera of trees in comparison to the chosen pines because this species is known to produce large amounts of pitch. One might ask if the combination of boring and acetone will not damage the tree. It must have some effect, but there has been no sign of damage in the growth or gas-exchange of five 30-cm diameter Scots pine trees treated similarly in 2014.

Another concern is the extent of cavitation in xylem cells near the borehole wall. Because the xylem functions under tension, cavitation must occur at least in the cells that were cut by the drill, and probably several cells deeper (Pfautsch, 2016; Wiedemann et al., 2016). In addition, there is a compartment formed around the wound, primarily to prevent microbial invasion (Morris et al., 2016). Constant evaporation in the borehole could dry up its surface if cavitation or compartmentation blocked the supply of water from the cavitated xylem vessels or tracheids. We could not detect any trend in the relative humidity within the borehole that would have indicated drying of the borehole. It is possible that even if the cell lumens are cavitated, water continued to diffuse through the cell walls toward the borehole wall. Continued wetting of the borehole surface was the reasoning behind model assumption 3.3, where borehole surface water tends toward isotopic equilibrium with xylem water because it is constantly renewed and mixed with the water evaporating at Craig-Gordon steady state. However, it seems that the wounding and its repair should eventually lead to isolation of the borehole water supply from the flowing xylem water, limiting how long a borehole can be used. We saw no evidence that we approached this limit in our experiments.

### Model Assumptions

The model was written such that it made three different assumptions about the evaporating water at the surface of the borehole. Model assumption 3.1 stated that the water vapor would equilibrate isotopically with xylem water in each segment of the borehole, and then be passed inward to equilibrate over the next segment, until the airstream left the borehole. This was based on the presumption that the xylem water pool is so much bigger than the borehole vapor pool that it would overwhelm the vapor signal on its passage through the stem. Note that this formulation contradicts the Craig-Gordon model (3.2) however, because the latter simply mixes water derived from the incoming water vapor with xylem water, disallowing any change in the borehole water vapor as it passes from one segment to the next. In contrast, model assumption 3.3 supposed that there is some mixing of the evaporating water at the borehole surface with the unenriched xylem water nearby. The mixing was set at different rates, from 0 to 1 per time step, and the model was then run as normal. This model drove the isotopic composition of the borehole vapor rapidly to equilibrium with the unenriched xylem sap if at least 35% of the borehole surface water was replaced by unenriched xylem water (Figure 6D). This could happen, for example, if 35% of the borehole was exposed to unenriched xylem water and the remainder was at Craig-Gordon steady state. We think it is likely that the mixing occurred due to diffusion toward and away from the evaporation/condensation sites, especially after the borehole humidity had reached saturation.

Continuous measurements of the isotopic composition of xylem sap describe an important middle ground between soil and leaves. Both surface soils and leaves undergo rapid fluctuations in isotopic composition. It is possible to predict their influence on transpiration, but it is difficult. Soils vary due to precipitation events and subsequent evaporation from the surface (Dubbert et al., 2013). Transpiration varies over the course of a day as the leaf water pools are enriched and then depleted by exchange with the atmosphere (Dubbert et al., 2014). The isotopic composition of xylem sap changes more slowly. It provides a direct integrated measure of the isotopic composition of water uptake from all potential water sources (Brinkmann et al., 2018, but see, e.g., Vargas et al., 2017). A continuous monitoring of xylem water isotopes might therefore enable to directly detect changes in water sourcing (e.g. during droughts), identify groundwater use of vegetation (compare Beyer et al., 2018) or investigate hydraulic redistribution. Likewise, the rapid diurnal fluctuations in the isotopic composition of transpired water necessarily converge, when integrated over a day, on the isotopic composition of the water flowing up the xylem (Dubbert et al., 2014). Thus, many of the complexities of leaf transpiration disappear in the xylem-sap data. What remains is the isotopic composition of the daily sum of root water uptake and hence transpiration.

Note that the model is based on a simple description of flow in the borehole, where a central core of moving air flows through a volume of still air. Within the still volume, water vapor diffuses to and from the borehole wall to and from the moving airstream. The model could be made more realistic and experimental tests of the flow conditions in the borehole could be conducted. We argue that the model is sufficient for the current purpose. That argument is supported by the agreement of the modeled and measured data.

## Conclusions

This study proves the potential utility of the stem borehole equilibration. The model leads us to suggest that the physics should be general, but it will of course be necessary to test the method in other species and other conditions. For example, angiosperm species with ring-porous wood, such as oak, might behave differently than the tracheid-bearing wood of pines. Comparisons to angiosperm species with and without tyloses and with varying hydraulic conductance would also be useful. The method appears to be accurate enough to measure the passage of small changes in natural abundance through the stems, for example after a precipitation event. In addition, we note that we were able to follow the passage of an isotopic label through tree stems. This might provide useful checks of *in situ* sap flow rates and a means of observing spatial patterns of water uptake in trees. *In situ* isotope methods offer an immense potential for describing and understanding the dynamic character of natural systems, which has been recognized by the scientific community as an urgent issue (e.g., Sprenger et al., 2016; Brantley et al., 2017; Penna et al., 2018). Combined data sets of depth-dependent soil water isotopes and xylem (transpiration) water isotopes will improve our understanding of ecosystems under changing environmental conditions and address several of the current shortcomings of traditional methods.

## Data Availability Statement

The datasets generated for this study are available on request to the corresponding author.

## Author Contributions

JM designed and conducted experiment 1. MB, MD, and KK designed and conducted experiment 2. MC wrote the model. All contributed to the writing.

### Conflict of Interest

The authors declare that the research was conducted in the absence of any commercial or financial relationships that could be construed as a potential conflict of interest.

## References

[B1] Barbeta A. Jones S. P. Clavé L. Wingate L. Gimeno T. E. Fréjaville B. (2018). Hydrogen isotope fractionation affects the identification and quantification of tree water sources in a riparian forest. Hydrol. Earth Syst. Sci. Dis. 23, 1–29. 10.5194/hess-2018-402

[B2] Barbeta A. Jones S. P. Clavé L. Wingate L. Gimeno T. E. Fréjaville B. (2019). Unexplained hydrogen isotope offsets complicate the identification and quantification of tree water sources in a riparian forest. Hydrol. Earth Syst. Sci. 23, 2129–2146. 10.5194/hess-23-2129-2019

[B3] Barr A. G. Black T. A. Hogg E. H. Griffis T. J. Morgenstern K. Kljun N. (2007). Climatic controls on the carbon and water balances of a Boreal Aspen forest, 1994–2003. Glob. Change Biol. 13, 561–576. 10.1111/j.1365-2486.2006.01220.x

[B4] Beyer M. Hamutoko J. T. Wanke H. Gaj M. Koeniger P. (2018). Examination of deep root water uptake using anomalies of soil water stable isotopes, depth-controlled isotopic labeling and mixing models. J. Hydrol. 566, 122–136. 10.1016/j.jhydrol.2018.08.060

[B5] Beyer M. Koeniger P. Gaj M. Hamutoko J. T. Wanke H. Himmelsbach T. (2016). A deuterium-based labeling technique for the investigation of rooting depths, water uptake dynamics and unsaturated zone water transport in semiarid environments. J. Hydrol. 533(Suppl.C), 627–43. 10.1016/j.jhydrol.2015.12.037

[B6] Brantley S. L. Eissenstat D. M. Marshall J. A. Godsey S. E. Balogh-Brunstad Z. Karwan D. L. (2017). Reviews and syntheses: on the roles trees play in building and plumbing the critical zone. Biogeosciences 14, 5115–5142. 10.5194/bg-14-5115-2017

[B7] Brinkmann N. Seeger S. Weiler M. Buchmann N. Eugster W. Kahmen A. (2018). Employing stable isotopes to determine the residence times of soil water and the temporal origin of water taken up by *Fagus sylvatica* and *Picea abies* in a temperate forest. New Phytol. 219, 1300–1313. 10.1111/nph.15255 29888480

[B8] Busch D. E. Ingraham N. L. Smith S. D. (1992). Water uptake in woody riparian phreatophytes of the Southwestern United States: a stable isotope study. Ecol. Appl. 2, 450–459. 10.2307/1941880 27759278

[B9] Craig H. Gordon L. I. (1965). “Deuterium and oxygen-18 variations in the ocean and the marine atmosphere,” in Stable Isotopes in Oceanographic Studies and Palaeotemperatures, ed E. Tongiorgi (Pisa: Lab. Geologia Nucleare), 9–130.

[B10] Cuntz M. Ogée J. Farquhar G. D. Peylin P. Cernusak L. A. (2007). Modelling advection and diffusion of water isotopologues in leaves. Plant Cell Environ. 30, 892–909. 10.1111/j.1365-3040.2007.01676.x 17617818

[B11] Dongmann G. Nürnberg H. W. Förstel H. Wagener K. (1974). On the enrichment of H218O in the leaves of transpiring plants. Rad. Environ. Biophys. 11, 41–52. 10.1007/BF01323099 4832051

[B12] Dubbert M. Cuntz M. Piayda A. Maguás C. Werner C. (2013). Partitioning evapotranspiration—testing the craig and gordon model with field measurements of oxygen isotope ratios of evaporative fluxes. J. Hydrol. 496, 142–153. 10.1016/j.jhydrol.2013.05.033

[B13] Dubbert M. Cuntz M. Piayda A. Maguás C. Werner C. (2014). Oxygen isotope signatures of transpired water vapor: the role of isotopic non-steady-state transpiration under natural conditions. New Phytologist 203, 1242–1252. 10.1111/nph.12878 24909361

[B14] Dubbert M. Werner C. (2019). Water fluxes mediated by vegetation: emerging isotopic insights at the soil and atmosphere interfaces. New Phytologist 221, 1754–1763. 10.1111/nph.15547 30341780

[B15] Ellsworth P. Z. Williams D. G. (2007). Hydrogen isotope fractionation during water uptake by woody xerophytes. Plant Soil 291, 93–107. 10.1007/s11104-006-9177-1

[B16] Evaristo J. Jasechko S. McDonnell J. J. (2015). Global separation of plant transpiration from groundwater and streamflow. Nature 525, 91–94. 10.1038/nature14983 26333467

[B17] Farquhar G. D. Cernusak L. A. Barnes B. (2007). Heavy water fractionation during transpiration. Plant Physiol. 143, 11–18. 10.1104/pp.106.093278 17210909 PMC1761995

[B18] Flanagan L. B. Ehleringer J. R. Marshall J. D. (1992). Differential uptake of summer precipitation among co-occurring trees and shrubs in a pinyon-juniper woodland. Plant Cell Environ. 15, 831–836.

[B19] Gaj M. Beyer M. Koeniger P. Wanke H. Hamutoko J. Himmelsbach T. (2016). *In situ* unsaturated zone water stable isotope (^2^H and ^18^O) measurements in semi-arid environments: a soil water balance. Hydrol. Earth Syst. Sci. 20, 715–731. 10.5194/hess-20-715-2016

[B20] Gaj M. Kaufhold S. Koeniger P. Beyer M. Weiler M. Himmelsbach T. (2017). Mineral mediated isotope fractionation of soil water. Rapid Commun. Mass Spectr. 31, 269–280. 10.1002/rcm.7787 27859754

[B21] Granier A. (1987). Evaluation of Transpiration in a Douglas-Fir Stand by Means of Sap Flow Measurements. Tree Physiol. 3, 309–20. 10.1093/treephys/3.4.309 14975915

[B22] Haverd V. Cuntz M. Griffith D. Keitel C. Tadros C. Twining J. (2011). Measured deuterium in water vapour concentration does not improve the constraint on the partitioning of evapotranspiration in a tall forest canopy, as estimated using a soil vegetation atmosphere transfer model. Agri. Forest Meteorol. 151, 645–654. 10.1016/j.agrformet.2011.02.005

[B23] Herbstritt B. Gralher B. Weiler M. (2012). Continuous *in situ* measurements of stable isotopes in liquid water. Water Resourc. Res. 48:W03601 10.1029/2011WR011369

[B24] Hogg E. H. Black A. T. den Hartog G. Neumann H. H. Zimmermann R. Hurdle P. A. (1997). A comparison of sap flow and eddy fluxes of water vapor from a boreal deciduous forest. J. Geophys. Res. 102, 28929–28937. 10.1029/96JD03881

[B25] Jarvis P. G. McNaughton K. G. (1986). “Stomatal control of transpiration: scaling up from leaf to region,” in Advances in Ecological Research, eds MacFadyen A. Ford E. D. (London: Academic Press), 1–49. 10.1016/S0065-2504(08)60119-1

[B26] Jasechko S. Sharp Z. D. Gibson J. J. Birks S. J. Yi Y. Fawcett P. J. (2013). Terrestrial water fluxes dominated by transpiration. Nature 496, 347–350. 10.1038/nature11983 23552893

[B27] Koeniger P. Marshall J. D. Link T. Mulch A. (2011). An inexpensive, fast, and reliable method for vacuum extraction of soil and plant water for stable isotope analyses by mass spectrometry. Rapid Commun. Mass Spectr. 25, 3041–3048. 10.1002/rcm.5198 21953958

[B28] Kulmatiski A. Beard K. H. Verweij R. J. T. February E. C. (2010). A depth-controlled tracer technique measures vertical, horizontal and temporal patterns of water use by trees and grasses in a subtropical Savanna. New Phytologist 188, 199–209. 10.1111/j.1469-8137.2010.03338.x 20561202

[B29] Lin G. Sternberg L. S. L. (1993). “Hydrogen isotopic fractionation by plant roots during water uptake in coastal wetland plants,” in Stable Isotopes and Plant Carbon- Water Relations, eds Ehleringer J. R. Hall A. E. Farquhar G. D. (San Diego, CA: Academic Press), 497–510.

[B30] Majoube M. (1971). Fractionnement en oxygene 18 et en deuterium entre l'eau et sa vapeur. J. Chimie Phys. 68, 1423–1436.

[B31] Marshall J. D. Waring R. H. (1986). Comparison of methods of estimating leaf-area index in old-growth douglas-fir. Ecology 67, 975–79.

[B32] Martín-Gómez P. Barbeta A. Voltas J. Peñuelas J. Dennis K. Palacio S.. (2015). Isotope-ratio infrared spectroscopy: a reliable tool for the investigation of plant-water sources? New Phytologist 207, 914–927. 10.1111/nph.13376 25790288

[B33] Moreira M. Z. da Sternberg L. S. L. Nepstad D. C. (2000). Vertical patterns of soil water uptake by plants in a primary forest and an abandoned pasture in the Eastern Amazon: an isotopic approach. Plant Soil 222, 95–107. 10.1023/A:1004773217189

[B34] Morris H. Brodersen C. Schwarze F. W. M. R. Jansen S. (2016). The parenchyma of secondary xylem and its critical role in tree defense against fungal decay in relation to the CODIT model. Front Plant Sci. 7:e1665. 10.3389/fpls.2016.01665 27881986 PMC5101214

[B35] Murray F. W. (1966). On the Computation of Saturation Vapor Pressure. Santa Monica: The RAND Corporation.

[B36] Ogée J. Cuntz M. Peylin P. Bariac T. (2007). Non-steady-state, non-uniform transpiration rate and leaf anatomy effects on the progressive stable isotope enrichment of leaf water along monocot leaves. Plant Cell Environ. 30, 367–387. 10.1111/j.1365-3040.2006.01621.x 17324225

[B37] Orlowski N. Frede H. G. Brüggemann N. Breuer L. (2013). Validation and application of a cryogenic vacuum extraction system for soil and plant water extraction for isotope analysis. J. Sensors Sensor Syst. 2, 179–193. 10.5194/jsss-2-179-2013

[B38] Orlowski N. Pratt D. L. McDonnell J. J. (2016). Intercomparison of soil pore water extraction methods for stable isotope analysis. Hydrol. Proces. 30, 3434–3449. 10.1002/hyp.10870

[B39] Penna D. Hopp L. Scandellari F. Scott Allen T. Benettin P. Beyer M. (2018). Ideas and perspectives: tracing terrestrial ecosystem water fluxes using hydrogen and oxygen stable isotopes—challenges and opportunities from an interdisciplinary perspective. Biogeosciences 15, 6399–6415. 10.5194/bg-15-6399-2018

[B40] Pfautsch S. (2016). Hydraulic anatomy and function of trees—basics and critical developments. Curr. Forestry Rep. 2, 236–248. 10.1007/s40725-016-0046-8

[B41] Poca M. Coomans O. Urcelay C. Zeballos S. R. Bodé S. Boeckx P. (2019). Isotope fractionation during root water uptake by acacia caven is enhanced by Arbuscular Mycorrhizas. Plant Soil. 441, 485–97. 10.1007/s11104-019-04139-1

[B42] Poyatos R. Granda V. Molowny-Horas R. Mencuccini M. Steppe K. Martínez-Vilalta J. (2016). SAPFLUXNET: towards a global database of sap flow measurements. Tree Physiol. 36, 1449–1455. 10.1093/treephys/tpw110 27885171

[B43] Priyadarshini K. V. R. Prins H. H. T de Bie S. Heitkönig I. M. A. (2016). Seasonality of hydraulic redistribution by trees to grasses and changes in their water-source use that change tree-grass interactions. Ecohydrology 9, 218–228. 10.1002/eco.1624

[B44] Quiñonez-Piñón R. M. Valeo C. (2017). Allometry of sapwood depth in five boreal trees. Forests 8:457 10.3390/f8110457

[B45] Rothfuss Y. Javaux M. (2016). Isotopic approaches to quantifying root water uptake and redistribution: a review and comparison of methods. Biogeosci. Dis. 14, 2199–2224. 10.5194/bg-2016-410

[B46] Rothfuss Y. Vereecken H. Brüggemann N. (2013). Monitoring water stable isotopic composition in soils using gas-permeable tubing and infrared laser absorption spectroscopy. Water Resourc. Res. 49, 3747–3755. 10.1002/wrcr.20311

[B47] Schulze E. -D. Mooney H. A Sala O. E Jobbagy E. (1996). Rooting depth, water availability, and vegetation cover along an aridity gradient in patagonia. Oecologia 108, 503–511. 10.1007/BF00333727 28307867

[B48] Simonin K. A. Roddy A. B. Link P. Apodaca R. Kevin Tu P. Hu J.. (2013). Isotopic composition of transpiration and rates of change in leaf water isotopologue storage in response to environmental variables. Plant Cell Environ. 36, 2190–2206. 10.1111/pce.12129 23647101

[B49] Soderberg K. Stephen Good P. Wang L. Caylor K. (2012). Stable isotopes of water vapor in the vadose zone: a review of measurement and modeling techniques. Vadose Zone J. 11:165 10.2136/vzj2011.0165

[B50] Sprenger M. Leistert H. Gimbel K. Weiler M. (2016). Illuminating hydrological processes at the soil-vegetation-atmosphere interface with water stable isotopes. Rev. Geophys. 54, 674–704. 10.1002/2015RG000515

[B51] Tor-ngern P. Oren R. Oishi A. C. Uebelherr J. M. Palmroth S. Tarvainen L.. (2017). Ecophysiological variation of transpiration of pine forests: synthesis of new and published results. Ecol. Appl. 27, 118–133. 10.1002/eap.1423 28052502

[B52] Turner D. P. Acker S. A. Means J. E. Garman S. L. (2000). Assessing alternative allometric algorithms for estimating leaf area of Douglas-fir trees and stands. For. Ecol. Manage. 126, 61–76. 10.1016/S0378-1127(99)00083-3

[B53] Vargas A. I. Schaffer B. Yuhong L. da Silveira Lobo Sternberg L. (2017). Testing plant use of mobile vs immobile soil water sources using stable isotope experiments. New Phytologist 215, 582–594. 10.1111/nph.14616 28556977

[B54] Volkmann T. H. M. Kühnhammer K. Herbstritt B. Gessler A. Weiler M. (2016). A method for *in situ* monitoring of the isotope composition of tree xylem water using laser spectroscopy. Plant Cell Environ. 39, 2055–2063. 10.1111/pce.12725 27260852

[B55] Volkmann T. H. M. Weiler M. (2014). Continual *in situ* monitoring of pore water stable isotopes in the subsurface. Hydrol Earth Syst Sci. 18, 1819–1833. 10.5194/hess-18-1819-2014

[B56] von Caemmerer S. Farquhar G. D. (1981). Some relationships between the biochemistry of photosynthesis and the gas exchange of leaves Planta 153, 376–387. 10.1007/BF00384257 24276943

[B57] Wershaw R. L. Friedman I. Heller S. J. Frank P. A. (1966). “Hydrogen isotopic fractionation of water passing through trees,” in Advances in Organic Geochemistry, International Series of Monographs on Earth Sciences, eds, Hobson G. D. Speers G. C. Inderson D. E. (New York, NY: Pergamon Press, 55–67.

[B58] West A. G. Goldsmith G. R Brooks P. D. Dawson T. E. (2010). Discrepancies between isotope ratio infrared spectroscopy and isotope ratio mass spectrometry for the stable isotope analysis of plant and soil waters. Rapid Commun. Mass Spectr. 24, 1948–1954. 10.1002/rcm.4597 20552579

[B59] West A. G. Goldsmith G. R Matimati I. Dawson T. E. (2011). Spectral analysis software improves confidence in plant and soil water stable isotope analyses performed by Isotope Ratio Infrared Spectroscopy (IRIS). Rapid Commun. Mass Spectr. 25, 2268–2274. 10.1002/rcm.5126 21755548

[B60] White J. W. C. Cook E. R. Lawrence J. R. Wallace B. S. (1985). The DH ratios of sap in trees: implications for water sources and tree ring DH ratios. Geochim Cosmochim Acta 49, 237–246. 10.1016/0016-7037(85)90207-8

[B61] Wiedemann A. Marañón-Jiménez S. Rebmann C. Herbst M. Cuntz M. (2016). An empirical study of the wound effect on sap flux density measured with thermal dissipation probes. Tree Physiol. 36, 1471–1484. 10.1093/treephys/tpw071 27587487

[B62] Yakir D. da Sternberg L. S. L. (2000). The use of stable isotopes to study ecosystem gas exchange. Oecologia 123, 297–311. 10.1007/s004420051016 28308584

